# New finding of *Toxorhina* (*Ceratocheilus*) limoniid fly in Eocene Baltic amber and the biogeographical context of the genus

**DOI:** 10.1038/s41598-022-23866-7

**Published:** 2022-11-12

**Authors:** Iwona Kania-Kłosok, Wiesław Krzemiński, Jacek Szwedo

**Affiliations:** 1 Department of Biology, Institute of Biology and Biotechnology, University of Rzeszów, Rzeszów, Poland; 2 Institute of Systematics and Evolution of Animals, Polish Academy of Sciences, Kraków, Poland; 3 Laboratory of Evolutionary Entomology and Museum of Amber Inclusions, Department of Invertebrate Zoology and Parasitology, Faculty of Biology, University of Gdańsk, 59, Wita Stwosza St., 80-308 Gdańsk, Poland

**Keywords:** Evolution, Zoology

## Abstract

Based on new fossil materials, a new species *Toxorhina* (*Ceratocheilus*) *christelius* sp. nov. has been described herein with complete documentation of drawings and photographs. Features such as wide spine on the gonocoxite differentiating the new species of *Toxorhina* were discussed. Finding new interesting fossil materials also allowed for providing an emended diagnosis and additional description of known Eocene species—*Toxorhina* (*Ceratocheilus*) *eridanus.* Comparison of chosen morphological features of fossil and recent representatives of the genus were given and key for fossil species of subgenus *Ceratocheilus* was introduced. Distribution of recent *Toxorhina* and evolutionary history of the genus were discussed. The results of research on fossil materials prove that the stratigraphic range of the subgenus *Ceratocheilus* and the genus *Toxorhina* goes back to the Eocene, there is no evidence of their existence on Earth before. Moreover, these insects were probably associated with a warm climate, they were found for example in Baltic amber, the deposits of which were formed mainly in a subtropical climate. The reach diversity of the genus *Toxorhina* of recent fauna is strictly observed in tropical zones around the world.

## Introduction

The Limoniidae genus *Toxorhina* Loew is represented in recent fauna by over 150 species and subspecies within three subgenera: *Ceratocheilus* Wesché, *Eutoxorhina* Alexander, *Toxorhina* Loew. Subgenus *Ceratocheilus* is currently the most speciose (85 species, vast majority in Old World), subgenus *Toxorhina* comprises 68 species (nearly 1/3 in New World) and subgenus *Eutoxorhina* is represented only in Australian and Oceanian region by three species (Table 1; Supplementary Table 1). Fossil record of *Toxorhina* is scarce and limited only to three species placed in subgenus *Ceratocheilus* and one unplaced to any subgenus. The *Toxorhina* (*Ceratocheilus*) *eridanus* Meunier was described based on inclusions in Eocene Baltic amber, the second one—*Toxorhina* (*Ceratocheilus*) *caucasiensis* Krzemiński and Freiwald is known from imprints in sediments of Miocene from Stavropol (Stavropol’ Kraï, Russia). *Toxorhina* (*Ceratocheilus*) *mexicana* was described from Miocene Mexican amber. Another species—*Toxorhina madagascariensis* Meunier was described from Holocene defaunation resin of Madagascar–, but it remains unplaced to any subgenus of *Toxorhina* (Table 2). Comparative studies of new fossil materials allowed to establish and describe a new species for science and provide a hypothesis about Eocene and post-Eocene stages of evolution of this fossil taxa rare in fossil record.

**Table 1:** Distribution of *Toxorhina* in recent fauna (biogeographic divisions after Holt et al.^1^; distribution and data according to Oosterbroek^2^).

Genus	Region	The number of species within the genus	Subgenera
*Toxorhina*	Afrotropical	**28**	*Ceratocheilus* (18) *Toxorhina* (10)
	Australian	**8**	*Ceratocheilus* (7) *Eutoxorhina* (1)
	Madagascan	**10**	*Ceratocheilus* (8) *Toxorhina* (2)
	Nearctic	**2**	*Toxorhina* (2)
	Neotropical	**6**	*Ceratocheilus* (3) *Toxorhina* (3)
	Oceanian	**31**	*Ceratocheilus* (16) *Eutoxorhina* (2) *Toxorhina* (13)
	Oriental	**36**	*Ceratocheilus* (20) *Toxorhina* (16)
	Panamanian	**34**	*Ceratocheilus* (10) *Toxorhina* (24)

Significant values are in bold.

**Table 2:** Information about all fossil species and specimens of *Toxorhina* examined and described.

Species	The number of specimens	Material examined (No.)	Sex	Type of material	Age/origin	Collection	Author and date of publication
*Toxorhina* (*Ceratocheilus*) *caucasiensis* Krzemiński and Freiwald	**2**	254/671 (+/−) **(holotype)**	?	Imprint	Middle Miocene/Stavropol (northern Caucasus USSR)	(PIN RAS)	Krzemiński and Freiwald^3^
		254/275 (+ /−)	?	Imprint	Middle Miocene/Stavropol (northern Caucasus USSR)	(PIN RAS)	Krzemiński and Freiwald^3^
*Toxorhina* (*Ceratocheilus*) *eridanus* Meunier	**10**	No data in the original work	♂	Inclusion	Eocene/Baltic amber	No data	Meunier^4^
		No data in the original work	♀	Inclusion	Eocene/Baltic amber	No data	Meunier^4^
		2532	♂	Inclusion	Eocene/Baltic amber	(GMUG) (coll. Klebs)	Alexander^5^ Kania^6^
		6964 VI 4991	♂	Inclusion	Eocene/Baltic amber	(GMUG)	Alexander^5^
		No data in the original work	♂	Inclusion	Eocene/Baltic amber	(ZMB)	Alexander^5^
		925/det. Alexander, 1925	♀	Inclusion	Eocene/Baltic amber	(NHMB)	Alexander^5^Kania^5^
		MP/3426	?	Inclusion	Eocene/Baltic amber	(ISEA PAS)	Kania^6^
		MP/3427	?	Inclusion	Eocene/Baltic amber	(ISEA PAS)	Kania^6^
		MP/4442	♂	Inclusion	Eocene/Baltic amber	(ISEA PAS)	Current publication
		CCHH 832–1	?	Inclusion	Eocene/Baltic amber	(SDEI) (coll. Ch. and H. W. Hoffeins)	Current publication
*Toxorhina* (*Ceratocheilus*) *mexicana* Kopeć, Kania and Krzemiński	**1**	PI II 1870 **(holotype)**	♀	Inclusion	Early Miocene/Mexican amber/Chiapas, Mexico	(NHM)	Kopeć, Kania and Krzemiński^7^
*Toxorhina madagascariensis* Meunier	**1**	No data in the original work	♀	Inclusion	Holocene/Madagascar Copal/Madagascar	coll. J. Evers jr. Altona-Barenfeld (Hambourg)	Meunier^8^
*Toxorhina* (*Ceratocheilus*) *christelius* sp. nov	**3**	MP/4441 **(holotype)**	♂	Inclusion	Eocene/Baltic amber	(ISEA PAS)	Current publication
		MP/4443	♂	Inclusion	Eocene/Baltic amber	(ISEA PAS)	Current publication
		CCHH 832-2	♂	Inclusion	Eocene/Baltic amber	(SDEI) (coll. Ch. and H. W. Hoffeins)	Current publication

Significant values are in bold.

## Results

### Systematic palaeontology

Order: Diptera Linnaeus

Infraorder: Tipulomorpha Rohdendorf

Family: Limoniidae Speiser

Subfamily: Limoniinae Speiser

Genus: *Toxorhina* Loew

Type species: *Toxorhina fragilis* Loew, by subsequent designation by Osten Sacken

Subgenus: *Ceratocheilus* Wesché

1910 *Ceratocheilus* Wesché, p. 358 (as genus)

Type species: *Ceratocheilus winnsampsoni* Wesché [= *Styringomyia cornigera* Speiser], by subsequent designation of Brunetti)

### ***Toxorhina*** (***Ceratocheilus***) ***eridanus*** Meunier

(Figures 1, 2, 3).

**Figure 1:**
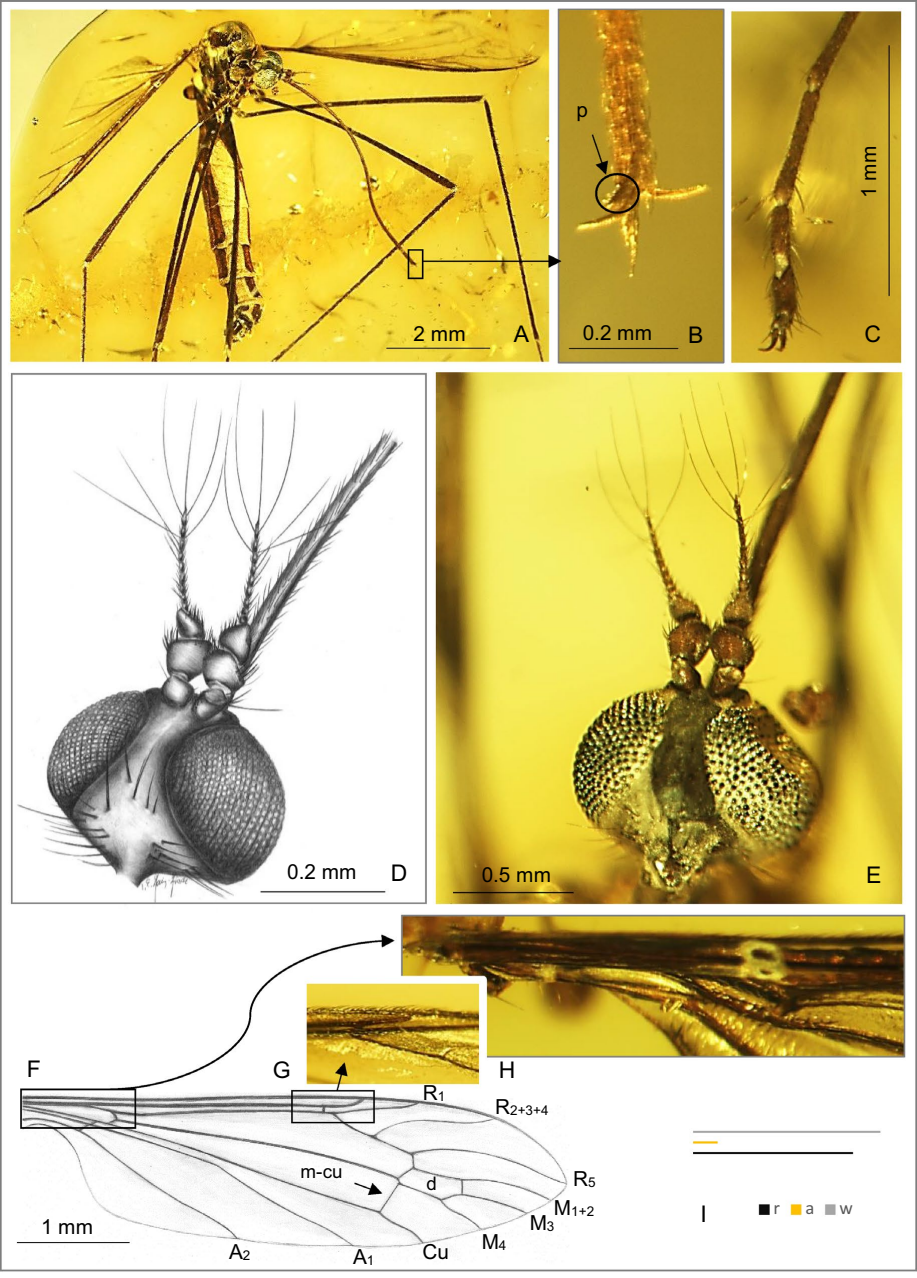
*Toxorhina* (*Ceratocheilus*) *eridanus* Meunier^5^ (Limoniidae), inclusions in Baltic amber, (**A**–**E**, **G**, **H**)—No. CCHH 832-1 (male) (coll. Ch. and H. W. Hoffeins), specimen from Senckenberg Deutsches Entomologisches Institut (SDEI) Müncheberg, Germany: (**A**) body, dorso-ventral view; (**B**) enlarged view of the tip of rostrum with palpus visible; (**C**) last tarsomeres; (**D**) drawing of head with part of rostrum, latero-dorsal view (reconstruction); (**E**) photograph of head with part of rostrum, latero-dorsal view; (**F**)—No. K2532 (male) (coll. Klebs), specimen from University of Göttingen, drawing of wing (reconstruction); (**G**) photograph of enlarged view of sc-r position; (**H**) photograph of enlarged view of base of wing; (**I**) relation between the length of rostrum (r), antenna (a) and wing (w). p—palpus.

**Figure 2:**
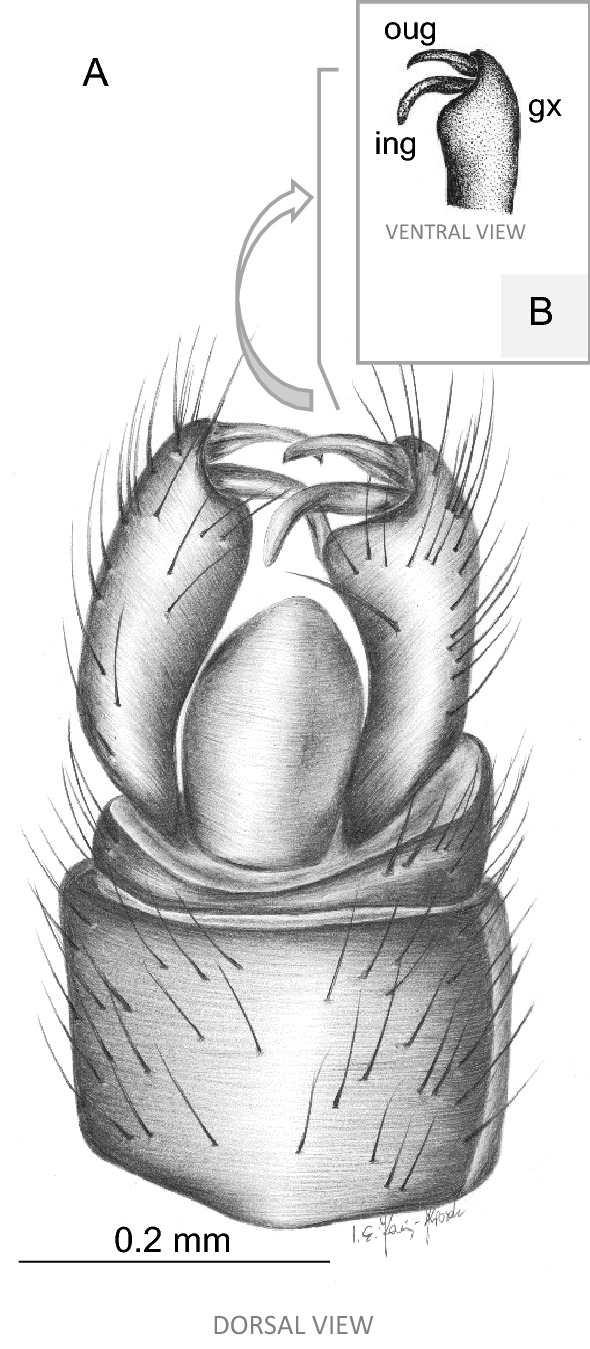
*Toxorhina* (*Ceratocheilus*) *eridanus* Meunier^4^ (Limoniidae), inclusions in Baltic amber, No. K2532 (male) (coll. Klebs), specimen from University of Göttingen: (**A**) drawing of hypopygium, dorsal view (reconstruction); (**B**) drawing of tip of gonocoxite without spine and gonostyles, ventral view (reconstruction). gx—gonocoxite; ing—inner gonostylus; oug—outer gonostylus.

**Figure 3:**
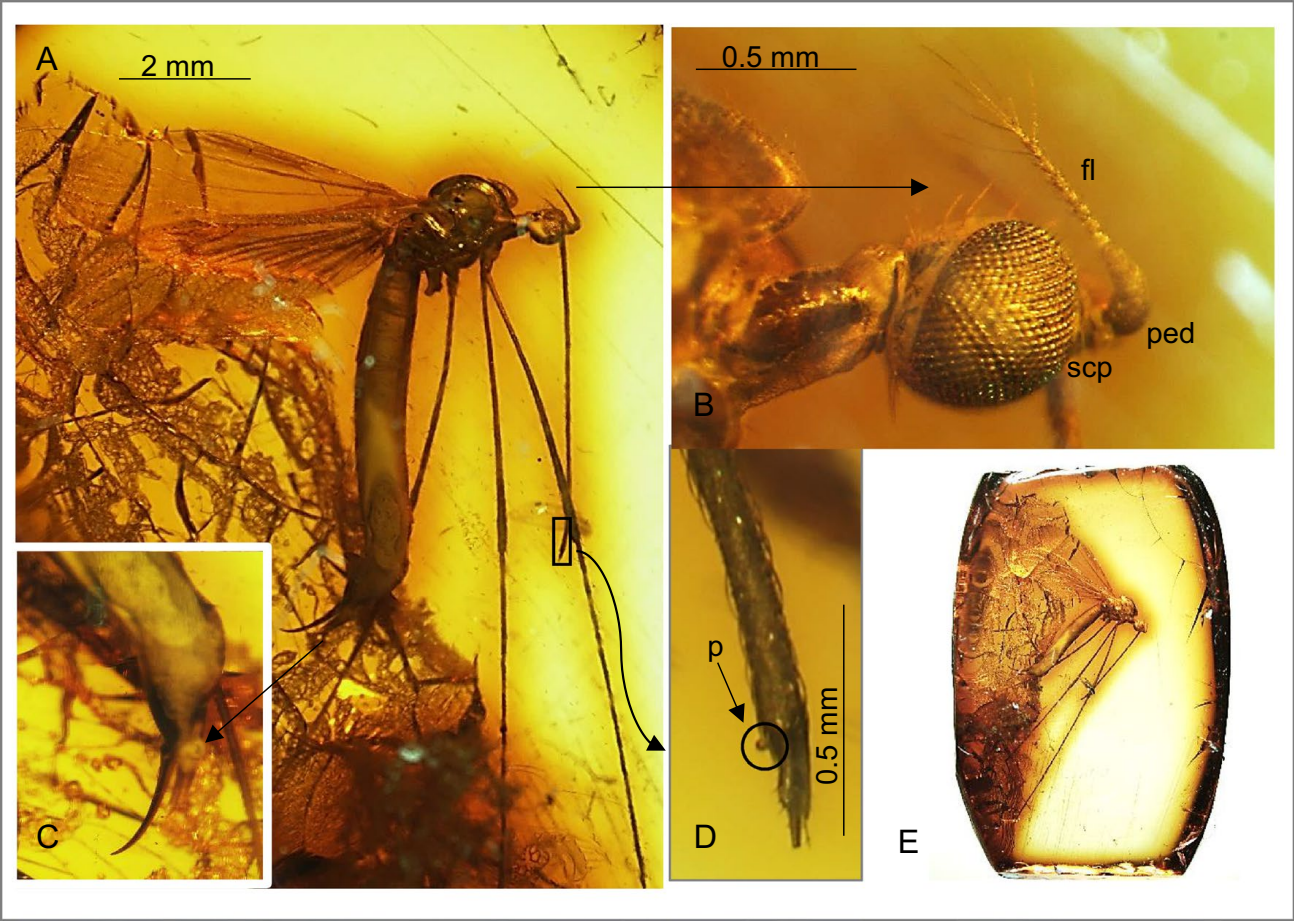
*Toxorhina* (*Ceratocheilus*) *eridanus* Meunier^4^ (Limoniidae), inclusion in Baltic amber, specimen from Natural History Museum Humboldt University, Berlin (NHMB) (female): (**A**) body, lateral view; (**B**) enlarged view of head, lateral view; (**C**) enlarged view of ovipositor; (**D**) enlarged view of the tip of rostrum with palpus visible; (**E**) amber piece with habitus in lateral view. fl—flagellum; scp—scape; ped—pedicel; p—palpus.

1917 *Ceratocheilus eridanus*—Meunier; p. 96–97, pl. 15, Figs. 67, 68, pl. 16, Fig. 72.

1931 *Ceratocheilus eridanus*—Alexander, p. 124, Figs. 168, 169.

1982 *Ceratocheilus eridanus*—Keilbach, p. 326.

1994 *Toxorhina* (*Ceratocheilus*) *eridanus*—Evenhuis, p. 87.

2015 *Toxorhina* (*Ceratocheilus*) *eridanus*—Kania, p. 85, 86, Figs. 26A–C, 34A–E.

*Material examined*. No. 925/det. Alexander, 1925 (female) Natural History Museum Humboldt University, Berlin (NHMB), No. K2532 (male) University of Gottingen (GMUG), coll. Klebs, No. CCHH 832-1 (male) Senckenberg Deutsches Entomologisches Institut (SDEI) Müncheberg, Germany, coll. Christel and Hans Werner Hoffeins; No. MP/4442 (male) Institute of Systematics and Evolution of Animals, Polish Academy of Sciences, Kraków, Poland (ISEA PAS).

*Additional description*. Body 6.48 long (male) (Figs. 1A–I; 2A,B)—7.43 long (excluding rostrum) (female) (Fig. 3A–E), head: head capsule 0.54 high (male), 0.72 long (male); rostrum 3.99 long (female)—4.38 long (male); antenna 0.67 long (1/0.09; 2/0.11; 3/0.06; 4/0.03; 5/0.03; 6/0.04; 7/0.04; 8/0.04; 9/0.04; 10/0.05; 11/0.06; 12/0.08) (male), 0.74 long (female), elongate setae of antenna 0.63 long (male); palpus very short; rostrum shorter than wing, antenna about 0.14 × of rostrum.

Thorax: wing 4.73 (female)—5.13 long, 0.85 (male)—1.35 (female) wide; Rs 0.44 mm (male) long; two last tarsomeres almost equal in length.

Abdomen: male terminalia—hypopygium 0.50 long, gonocoxite 0.37 long, ovipositor 1.46 long.

*Remarks*. Even though, Meunier^3^ provided a brief description of the male and female of *Toxorhina* (*Ceratocheilus*) *eridanus* and the species was reviewed by Alexander^4^ based on males specimens, specified clear diagnostic characters were given in 2015^5^. New fossil materials allow to provide additional description of the species.

### *Toxorhina* (*Ceratocheilus*) *christelius* sp. nov.

(Figures 4, 5).

**Figure 4:**
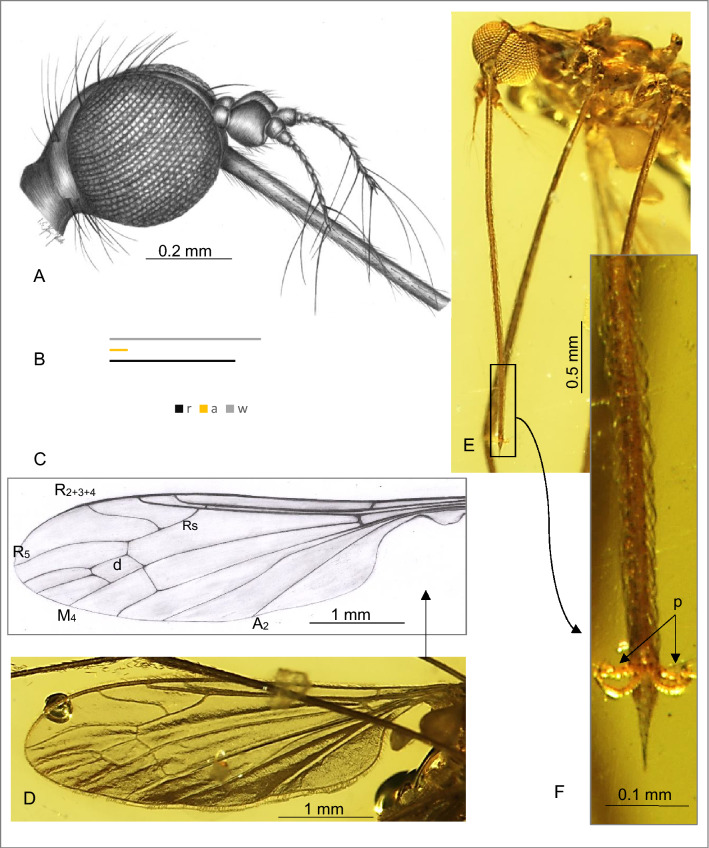
*Toxorhina* (*Ceratocheilus*) *christelius* sp. nov. (Limoniidae), inclusion in Baltic amber, (**A**) No. CCHH 832-2 (male), (coll. Ch. and H.W. Hoffeins), specimen from Senckenberg Deutsches Entomologisches Institut (SDEI) Müncheberg, Germany: (**A**) drawing of head with part of rostrum, lateral view (reconstruction); (**B**) relation between the length of rostrum (r), antenna (a) and wing (w); (**C**–**F**) No. MP/4441 (male), holotype, specimen from Institute of Systematics and Evolution of Animals, Polish Academy of Sciences, Kraków, Poland (ISEA PAS): (**C**) drawing of wing; (**D**) photograph of wing; (**E**) head, frontal view; (**F**) tip of rostrum with palpi well visible.

**Figure 5:**
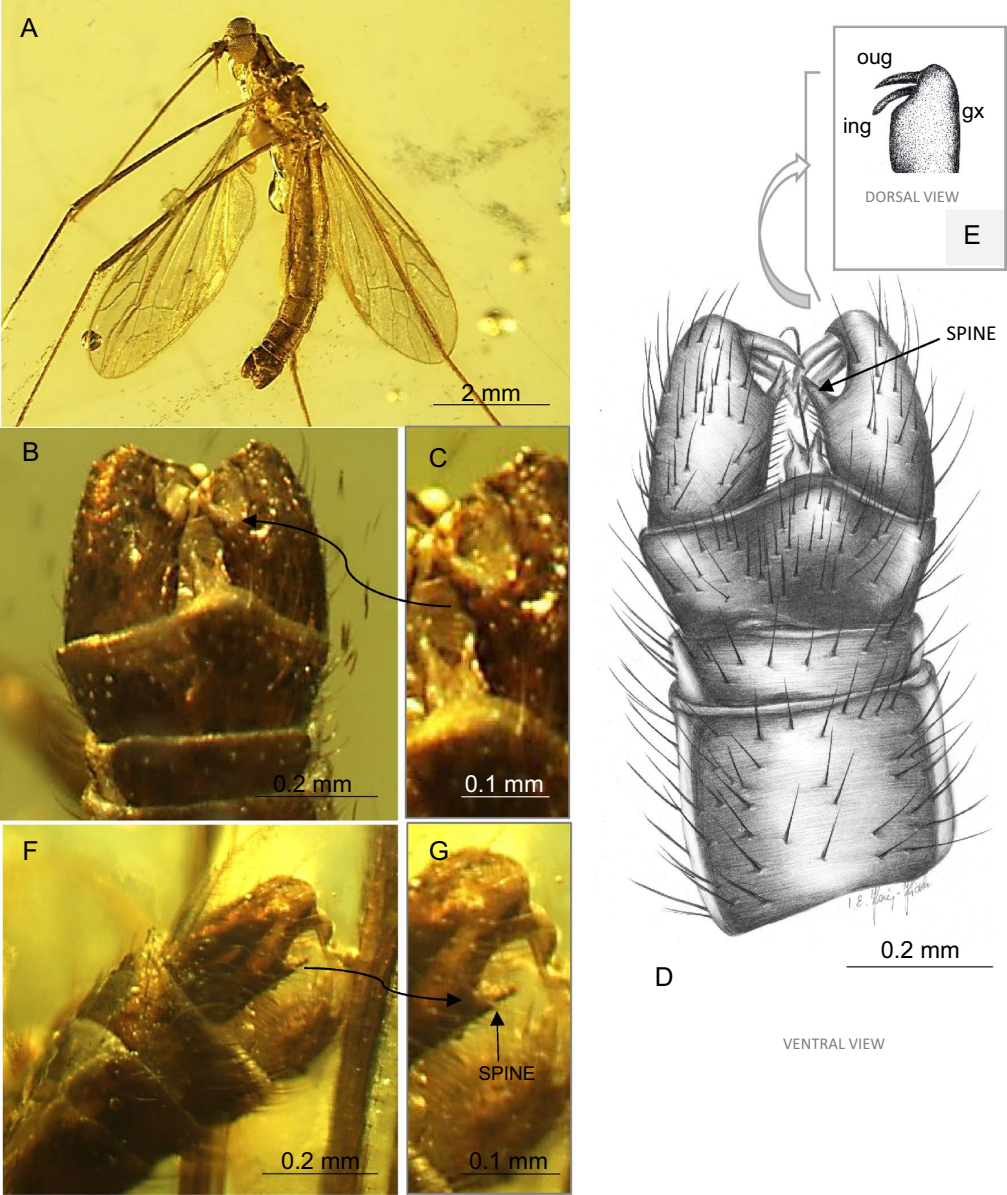
*Toxorhina* (*Ceratocheilus*) *christelius* sp. nov. (Limoniidae), inclusion in Baltic amber, (**A**–**E**) No. MP/4441 (male), holotype, specimen from Institute of Systematics and Evolution of Animals, Polish Academy of Sciences, Kraków, Poland (ISEA PAS): (**A**) body, ventral view; (**B**) photograph of hypopygium with spine on gonocoxite, ventral view; (**C**) enlarged view of spine on gonocoxite; (**D**) drawing of hypopygium—gonocoxite with spine, ventral view (reconstruction); (**E**, **F**) No. MP/4443 (male), holotype, specimen from Institute of Systematics and Evolution of Animals, Polish Academy of Sciences, Kraków, Poland (ISEA PAS): (**F**) photograph of hypopygium, latero-ventral view; (**G**) photograph of enlarged view of spine on gonocoxite, latero-ventral view. Abbreviations: the black arrow indicates spine.

*Diagnosis*. Crossvein sc-r situated before fork of Rb; Rs elongate, longer than basal section of R_5_ between its origin and point of contact with r-m; R_2+3+4_ about twice as long as Rs, reaching wing margin in approximately 0.3 × distance from tip of R_1_ to tip of R_5_; tip of R_2+3+4_ just beyond level of d-cell; d-cell almost rhomboidal; m-cu at or just before fork of Mb on M_1+2_ and M_3+4_; distance between M_1+2_ and M_3_ narrow, branch M_3_ and M_4_ about twice as wide as distance of M_1+2_ and M_3_ (in the widest area); gonocoxite with distinctly distinguished, wide, huge spine of internal part of hypopygium.

*Etymology.* The specific name is dedicated to Christel Hoffeins (Hamburg, Germany), the owner of amber inclusions collections and the expert of inclusions in Baltic amber.

*Material examined*. Holotype No. MP/4441 (male) Institute of Systematics and Evolution of Animals, Polish Academy of Sciences, Kraków, Poland (ISEA PAS). Additional material: No. MP/4443 (male) (ISEA PAS), No. CCHH 832-2 (male) Senckenberg Deutsches Entomologisches Institut (SDEI) Müncheberg, Germany (the collection of Christel and Hans Werner Hoffeins).

*Description*. Body (Figs. 4A–F; 5A–D) 4.41–6.03 long (excluding rostrum), brown, wings without color pattern, pterostigma absent.

Head: head capsule 0.47 high, 0.54 wide, rostrum 2.86–3.81 long; antenna about 0.55 long (1/0.06; 2/0.10; 3/0.06; 4/0.02; 5/0.02; 6/0.03; 7/0.03; 8/0.03; 9/0.03; 10/0.04; 11/0.05; 12/0.08); approximately as long as head, scape cylindrical, narrow, short, pedicel large, massive, widened in midlength, slightly wider than long, first flagellomere large, wider than long, much wider than other flagellomeres but distinctly smaller than pedicel, remaining flagellomeres cylindrical, longer than wide, flagellomeres become slender and more elongate to apex of antenna, last flagellomere narrow, on the last three flagellomeres occur very elongate setae according to pattern: eight flagellomere with one very elongate setae, the last two flagellomeres with three very elongate setae, longer than half length of antenna, approximately 0.32 long; additionally on flagellomeres are presented two not very elongate setae, much shorter than length of flagellomeres bearing them and numerous very short setae; palpus very short, 0.04 long, about 0.125 × of rostrum; rostrum shorter than wing, antenna about 0.16 × of rostrum.

Thorax: scutellum widened in midlength; wing 3.67–4.77 long, 1.06–1.28 wide; Rs 0.62 long; vein M_3_ 1.5 × the length of d-cell; d-cell twice as long as wide, 0.33 long; haltere with narrow, elongate stem slightly longer than knob.

Abdomen: male terminalia—hypopygium 0.43–0.49 long, gonocoxite, approximately twice as long as wide with wide, massive spine on internal part of ventral side of gonocoxite; outer gonostylus elongate, inner gonostylus narrow, sharpened at their end, slightly widened at base, generally tiny and slender.

*Comparison*. *T.* (*C.*) *christelius* sp. nov. differ from *T.* (*C.*) *eridanus* especially by morphology of hypopygium. Gonocoxite of a new species comes with distinctly distinguished, wide, huge spine on internal part of ventral side of gonocoxite, clearly visible from ventral side of hypopygium. In *T.* (*C.*) *eridanus* spines on gonocoxite does not occur, the gonocoxite of *T.* (*C.*) *eridanus* is only slightly widened distally. Moreover, tip of vein R_2+3+4_ in *T.* (*C.*) *christelius* sp. nov. is situated at the level of fork of M_1+2_ or just beyond, in *T.* (*C.*) *eridanus* tip of R_2+3+4_ is situated far beyond level of d-cell, while for example in *T.* (*C.*) *caucasiensis* vein R_2+3+4_ is rather short and its tip is situated approximately at the level of half d-cell. In *T.* (*C.*) *mexicana* R_2+3_ is very short, equal in length to Rs, while in all known from fossil record species is longer. In *T.* (*C.*) *eridanus* and *T.* (*C.*) *christelius* sp. nov. sc-r occur before or at fork of Rb, in *T.* (*C.*) *caucasiensis* beyond this bifurcation. In addition, the distance between veins M_1+2_ and M_3_ in the *T.* (*C.*) *christelius* sp. nov. is narrow, what is well visible at the level of vein m-m, in *T.* (*C.*) *caucasiensis*, *T.* (*C.*) *eridanus* or *T.* (*C.*) *mexicana* veins M_1+2_ and M_3_ are widely separated. In contrast to *T.* (*C.*) *madagascariensis*, basal part of d-cell of *T.* (*C.*) *madagascariensis* is narrowed, in new species is distinctly wide.

### Key to fossil species of genus *Toxorhina*

Basal part of d-cell rectangular.......2.
  –. D-cell narrowed at its base......***T. madagascariensis*** Meunier
2. Vein R_2+3+4_ longer than Rs; tip of R_2+3+4_ beyond the level of fork of Mb......3.
  –. Vein R_2+3+4_ very short, equal in length of vein Rs; tip of R_2+3+4_ before or at the level of fork of Mb......***T.* (*C.*) *mexicana*** Kopeć, Kania and Krzemiński (Fig. 6G,H)

**Figure 6:**
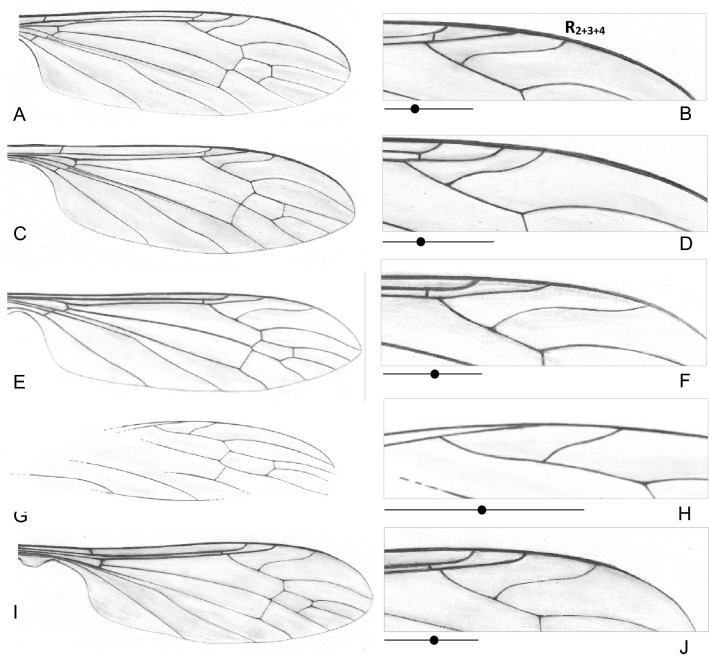
Wing venation of fossil *Toxorhina* (*Ceratocheilus*): (**A–D**) *Toxorhina* (*Ceratocheilus*) *caucasiensis* Krzemiński and Freiwald^6^; (**E**, **F**) *Toxorhina* (*Ceratocheilus*) *eridanus* Meunier^5^, (**G**, **H**) *Toxorhina* (*Ceratocheilus*) *mexicana* Kopeć, Kania, Krzemiński, 2016 (after Kopeć et al*.*^7^), (**I**, **J**) *Toxorhina* (*Ceratocheilus*) *christelius* sp. nov. (**B**, **D**, **F**, **H**, **J**)—enlarged view of part of wing with position of vein R_2+3+4_ with a diagram showing the position (black point) of the Rs bifurcation.
3. Tip of R_2+3+4_ positioned at the level of d-cell; sc-r occurs at or before the level of fork of Mb; the distance between tips of R_1_ and R_2+3+4_ rather wide, about 0.3× the distance between tips of R_2+3+4_ and R_5_; tip of A_1_ just before of m-cu and of fork of Mb level......4.
  –. Tip of R_2+3+4_ positioned before the level of d-cell; sc-r occurs beyond the level of fork of Mb; the distance between tip of R_1_ and R_2+3+4_ very short, about 0.2× the distance between tips of R_2+3+4_ and R_5_; tip of A_1_ at or just beyond of m-cu and of fork of Mb level......
  ***T.* (*C.*) *caucasiensis*** Krzemiński and Freiwald (Fig. 6A–D)
Wide spine present on gonocoxite; R_2+3+4_ separates Rs beyond half the length of the distance from fork of Rb to the point of connection of cross-vein r-m; the distance between M_1+2_ and M_3_ narrow, about twice as narrow as branch of M_3_ and M_4_; d-cell almost rhomboidal......
  ***T.* (*C.*) *christelius*** sp. nov. (Figs. 4; 5; 6I,J)
  –. Gonocoxite without spine; R_2+3+4_ separates Rs before half the length of the distance from fork of Rb to the point of connection of cross-vein r-m; the distance between M_1+2_ and M_3_ wide, about as wide as branch of M_3_ and M_4_ or slightly wider; d-cell almost rectangular;......
  ***T.* (*C.*) *eridanus*** Meunier (Fig. 6E,F)

## Discussion

The subgenus *Ceratocheilus* is well distinguishable from two other subgenera of *Toxorhina* by morphology of wing with two branches of Rs reaching the wing margin and the presence of corniculus (soft, pale, triangular sac on anterior vertex). In subgenera as *Toxorhina* and *Eutoxorhina* single branch of Rs reaches the wing margin^9^, therefore in these subgenera venation tends to be reduced and simplified (three medial veins reaching the margin in subgenus *Toxorhina* and two medial veins reaching margin in *Eutoxorhina*). The antennae of *Toxorhina *sensu lato are peculiar, in the male 12-segmented, with several basal antennomeres united into truncated, conical fusion-segment, the flagellar antennomeres, except the last two, glabrous, the outer pair with very long and conspicuous verticils. In the female the antenna is apparently 14 or l5 segmented, with the outer four or five flagellomeres provided with elongate verticils. Male hypopygium is peculiar and variable, in general basistyle (gonocoxite) bears or not an apical spine; dististyles (gonostyles) are variously shaped; aedeagus deeply to profoundly bifid, more or less resembling a tuning fork; ovipositor with the cerci long and slender, gently upcurved to the acute tips; hypogynal valvae shorter, compressed. All subgenera of *Toxorhina* are characterized by elongated rostrum, exceeding half of body length, or the entire body or wing length, bearing the reduced mouthparts at the tip. The biology of the group is known very little, adults use the long mouthparts to probe a variety of flowering plants for nectar^10,11^. The immature stages were found in slimy, well rotten wood and branches, under leaf litter, in the saturated soil of swamps and in the liquid between fibers in petioles of rotten palm fronds^11,12^. Adults found at the salt marshes^13^ also support such kind of habitat for immatures. The species of the genus *Toxorhina* are believed to represent strongly an ‘equatorial’ group^2^ (Table 1, Supplementary Table 1). Subgenus *Ceratocheilus* covers 18 species recorded in Afrotropical region, eight in the Madagascan region, 20 in Oriental, seven in Australian and 16 in Oceanian, two in Novozealandic, ten in Panamanian and three species in Neotropical regions. The subgenus *Ceratocheilus* is absent in Holarctic regions, also not recorded in Saharo-Arabian and reaching borders of Sino-Japanese regions. Subgenus *Toxorhina* presents a different pattern, with 24 species in the Panamanian region but with two species reaching further north to Nearctic region and a three more south-east to Neotropical region, 16 species recorded in Oriental region, 13 in Oceanian, ten in Afrotropical and two in Madagascan regions. Subgenus *Eutoxorhina* is restricted to single species in Australian region and two in Oceanian region. The data available suggest more restricted distributional patterns of *Ceratocheilus* in tropical-subtropical zones. Some species from northern parts of Oriental region—like e.g. *Toxorhina* (*Ceratocheilus*) *bistyla* Alexander, *T*. (*C*.) *brevifrons* (Brunetti), *T*. (*C*.) *fulvicolor* Alexander, *T.* (*C.*) *huanglica* Zhang, Li and Yang, *T*. (*C*.) *omnifusca* Zhang, Li and Yang are distributed in higher, montane habitats, between 1000 and 3500 m a.s.l.^9,14,15^, these from more southern parts of Oriental regions seem to present similar preferences. Montane habitats seems to be favorable also for Panamanian species of subgenus *Ceratocheilus*, the same could be inferred for Oceanian (mainly known from New Guinea) species. Afrotropical species seem to be present in various habitats from highlands to lowland marshlands, the latter seems to be also habitat for Neotropical species. Regarding the scarce fossil record of the subgenus *Ceratocheilus*—the oldest known species come from the Eocene from Baltic amber—*T*. (*C*.) *eridanus* Meunier and described above *T*. (*C*.) *christelius* sp. nov. These finding gave some additional data to discussion on reconstructions of the palaeohabitats of the Baltic amber forests. Some montane species of insects were reported from Baltic amber, as well as these with modern relatives present in marshlands and other humid habitats^16^.

**Figure 7:**
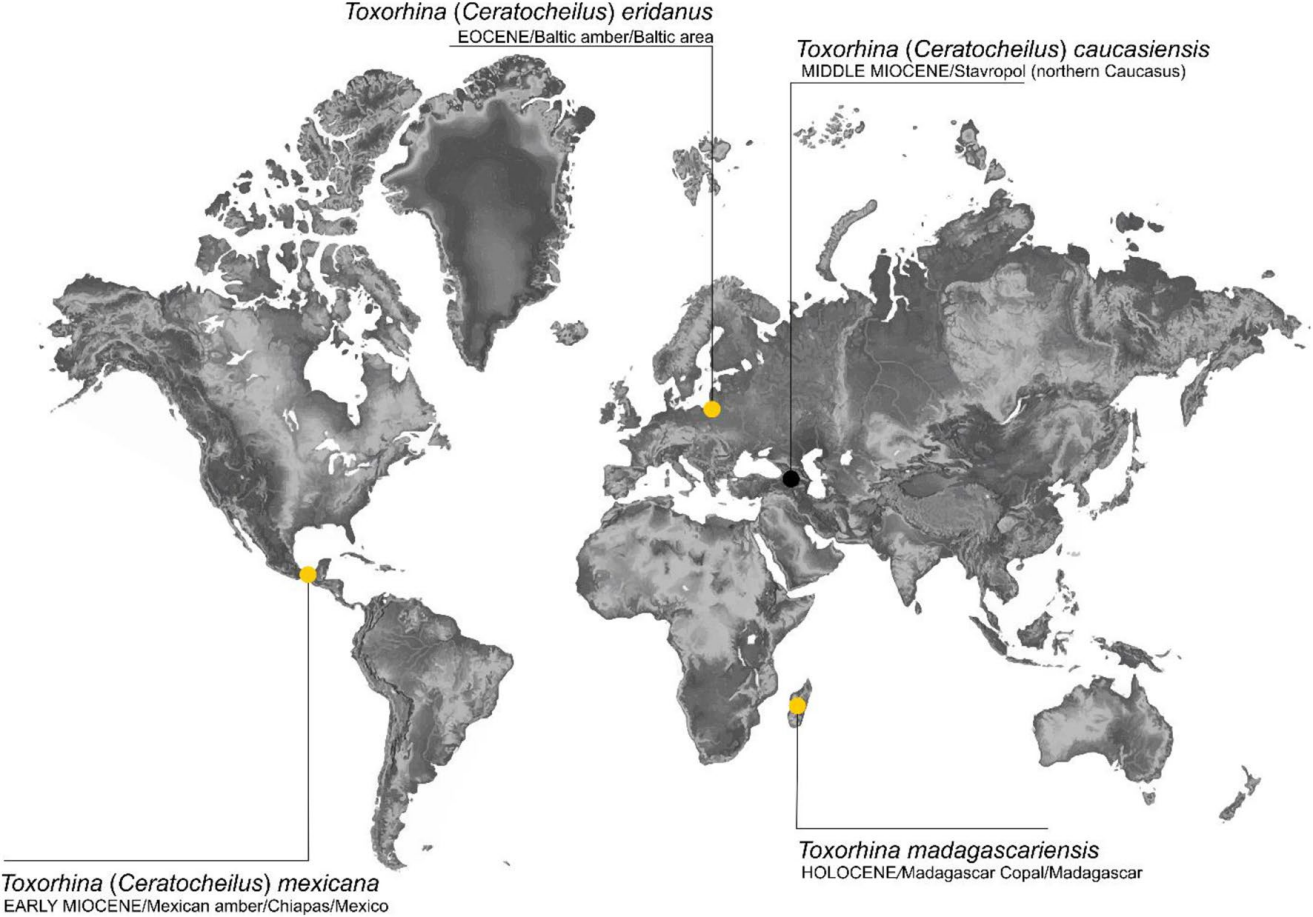
Map of the distribution of know localities of the representatives of the genus *Toxorhina*.

Miocene findings of the fossils ascribed to the subgenus *Ceratocheilus* are limited. A female named *Toxorhina* (*Ceratocheilus*) *mexicana* Kopeć, Kania and Krzemiński was described from Lower Miocene (Burdigalian) amber of Chiapas (Mexico) (Fig. 7). The reconstruction of the Mexican amber forest shows it to be close to modern lowland tropical forest, especially to mangrove formation^17–20^.

Another species comes from Middle Miocene, ca. about 13 Ma (Late Chokrakian; Serravalian) deposits of Stavropol (Stavropol’ Region, Russia). The aquatic groups (dragonflies, caddisflies, mayflies, aquatic beetles, bugs, some dipterans) compose 30% of all found insect imprints (of a total some 3000 specimens)^21^. The depositional conditions reconstructed presents mixture of lentic and lotic habitats, possibly a lagoon, a coastal freshened area, with periodical inflow of fresh water from nearby rivers, the presence of oxbow lakes and creeks^21^. Such habitats correspond quite well with known requirements of recent species of the subgenus *Ceratocheilus*, at its immature stages. Former investigations^22,23^ indicated climatic conditions shifting from warm humid to tropical during the time of deposition, with several biocenosis from littoral and coast plants associations, submerged and waterside plant associations, floodplain forests, deciduous-evergreen forests, to light, dry pine forests and light, dry deciduous forests and steppe type shrubs. Such variable habitats seems to offer a good food-source for imagines of flies.

The existence of a warmer and more equable climate in the Eocene and Miocene is now an indisputable fact^24^. These times were probably suitable for ancestors of recent taxa of the subgenus *Ceratocheilus* to spread and migrate in search of appropriate habitats. During the Eocene Temperature Optimum an exchange processes between the faunas of North America and the Western Palearctic was possible due to existence of land bridges^24,25^. Subsequent cooling, a decrease in the CO_2_ and oxygen concentrations in the atmosphere, aridization, etc., during the Oligocene decreased availability of appropriate habitats and farther changes and biogeographic shifts were caused by the next Miocene Climate Optimum^26^. The data available for the genus *Toxorhina* and its subgenera are very scarce, however patterns observed among other insects, e.g. in thermophilic ants^23^ or Lophopidae planthoppers^27–29^ could be a good reference for reconstruction of biogeographic scenario of these flies. Ancestral forms of *Toxorhina* most probably diversified during the warming periods of the Eocene (Paleocne-Eocene Thermal Maximum and Eocene Climatic Optimum) in the warm, humid areas of Europe in Thulean and western part of Boreal regions^30^, as documented by fossils from Baltic amber. The Limoniidae genera *Elephantomyia* Osten Sacken and *Helius* Lepeletier and Serville have rostrum elongate as in *Toxorhina* and its subgenera, in the genus *Geranomyia* Haliday mouth parts of are strongly elongate, but rostrum short^9,31–33^. The phylogenetic position of the genus *Toxorhina* within Limoniinae remains unclear, but it could be assumed that it separated somewhere in Late Cretaceous with diversification and spread to various habitats of nectar producing angiosperms^34,35^. Tendency to elongation of rostrum is observed in *Helius* since the Cretaceous with wide diversity in the Eocene^32,33^, and similar disparity pattern could be assumed for ancestors of *Toxorhina*. In addition, the morphological disparity of the *Toxorhina* is exposed also in venation patterns resulting in taxonomic separation to subgenera. The plesiomorphic condition in Limoniinae is placement of veinlet m-cu behind fork of Mb, in the Eocene *Ceratocheilus* it is placed at fork of Mb and in the Mid-Miocene taxa it is place anterior of fork or at fork of Mb. Regarding distributional patterns it could be assumed that ancestors of modern subgenus *Toxorhina* as well as ancestors of modern *Ceratocheilus* rapidly spread in the northern Hemisphere, crossing Atlantic Ocean in the Paleocene to earliest Eocene through De Geer and Thulean land bridges existing at these times^24,25^. Eastward spreading of ancestors of all subgenera probably took place in the Palaeocene–Eocene as well, but with Oligocene and Miocene climatic changes, colling and drying and decline of megathermal forest^36,37^, ancestors of modern representatives of subgenera *Toxorhina* and *Ceratocheilus* became relic in montane megathermal localities^2^. These changes together with tectonic events^27^ in the area resulted in further diversification and ancestors of modern subgenus *Eutoxorhina* reached Australia, Fiji and New Caledonia^2^. In the New World ancestors of both subgenera *Toxorhina* and *Ceratocheilus* could be assumed to migrate to suitable habitats with tectonic and climatic changes^37–39^, to montane and swampy areas of Central and South America^2^. Here these forms diversified and adapted to local conditions resulting in modern diversity (Table 1, Supplementary Table 1).

These disparity events resulted in the differences of the relation between the length of rostrum and wing species of recent fauna of *Ceratocheilus*. In *Toxorhina* (*Ceratocheilus*) *fulvicolor* Alexander described from India rostrum is shorter than wing as in *Toxorhina* (*Ceratocheilus*) *contractifrons* (Edwards) known from Malaysia or *Toxorhina* (*Ceratocheilus*) *omnifusca* described from China. In fossil representatives of subgenus e.g. in *T.* (*C.*) *eridanus* and newly described species *T.* (*C.*) *christelius* sp. nov. wing is longer than rostrum. The most obvious features that differentiate of some fossil species of *Thoxorhina* are morphology of hypopygium, but especially shape, position and length of vein R_2+3+4_, length of Rs, and position of tips of Sc and R_1_ (Fig. 6A–E). In *T.* (*C.*) *christelius* sp. nov. wide, massive spine occur on gonocoxite. For example in *T.* (*C.*) *caucasiensis* Rs is very short and R_2+3+4_ separate *sector radii* before half the length of Rs and basal section of R_5_ combined, while in *T.* (*C.*) *eridanus* R_2+3+4_ separate Rs beyond half the length of this section. Both, in recent and fossil fauna of *Ceratocheilus* morphology of antenna is not very differentiated, 12-segmented antenna with very elongate setae on the last segments, usually longer than half the length of antenna*.*

The most characteristic feature for subgenus *Ceratocheilus* is elongate rostrum, similarly to a craneflies belonging to the genus *Helius* Lepeletier and Serville or *Elephantomyia* Osten Sacken. The group of Limoniinae Speiser which is characterized by elongate rostrum, distinctly differ by other morphological feature, for example last palpomere of the genus *Helius* is very elongate, sometimes reaching the length of the previous three, while in representatives of subgenus *Ceratocheilus* of the genus *Toxorhina* palpus is reduced, similarly to this of *Elephantomyia*. Moreover, relations of the length of head or body, antenna, palpus and rostrum distinctly differ in these three subgenera. Elongate rostrum of *Helius* perhaps was used for a specific food spectrum, it was probably the nectar of flowers^33,40–44^. These three groups of insects are probably closely related, as it was suggested based on molecular data and fossil analysis the genera as *Helius*, known since early Cretaceous period, and much younger *Elephantomyia*^44,45^. The discovery of new *Ceratochelius* in Eocene Baltic amber contributes to a better understanding of the diversity and disparity of the genus *Toxorhina* and also subfamily Limoniinae, and it is important for further research on the evolution of this group of insects. So far, only seven specimens of *Toxorhina* (*Ceratocheilus*) from Baltic amber were known, new materials (seven not known so far specimens) give us interesting information, especially about morphology of gonocoxite, for example in new species *T.* (*C.*) *christelius* sp. nov. characteristic wide spine on gonocoxite is present, while in *T.* (*C.*) *eridanus* this structure is absent.

## Conclusions

The finding of more fossils of *Toxorhina* should make it possible to understand the patterns of morphological evolution and disparity associated with the adaptation of these flies to their mode of life, present conclusions about their (palaeo)biogeography, and evolution of biocoenoses inhabited by these insects.

## Material and methods

### Taxonomic treatments

To avoid a lengthy list of references dealing with scientific names detailed taxonomic treatment and chresonymy is given in the supplementary materials.

### Geological context

The age range of all Baltic amber bearing strata possibly cover 48 to 23 million years, but it is still controversial though has been a matter of debate for many years^46–55^. Based on pollen, spores and phytoplankton of the amber embedding layer, the Blue Earth the most current state of knowledge is that it is of Priabonian age, estimated between 38 and 34 million years old^47,52^. The age of Baltic amber has also been determined to approximately 47–41 Ma^4^, but the reliability of the methods used in his study have been questioned due to contaminations that can lead to older age estimations^48–51,53,54^.

### Specimen repository

The study was based on seven inclusions of the subgenus *Ceratocheilus* (Limoniidae: *Toxorhina*) preserved in Baltic amber. All the studied specimens are deposited in public institutions. The material examined is deposited in Senckenberg Deutsches Entomologisches Institut (SDEI) Müncheberg, Germany (the collection of Christel and Hans Werner Hoffeins) (two inclusions), Natural History Museum Humboldt University, Berlin (NHMB) (one specimen), University of Göttingen (GMUG) (one specimen), Institute of Systematics and Evolution of Animals, Polish Academy of Sciences, Kraków, Poland (ISEA PAS) (three specimens).

## Methods

The pieces of amber were examined using a Nikon SMZ 1500 stereomicroscope equipped with a Nikon DS-Fi1 camera, and the measurements were taken with NIS-Elements D 3.0 software in the lab of the Department of Biology, University of Rzeszów. Measurements of individual parts of the body were given only when the measured morphological structures were not distorted. All measurements are given in mm. Measurements of the vein M_3_ were taken from the point of connection of vein m-m with vein M_3_ to the margin of wing, measurements of the discal cell were taken from the proximal to distal ends of the d-cell. The length of hypopygium was measured from the posterior margin of tergite IX to the apex of gonocoxite. Basal section of R_5_ was mentioned as the length from fork of Rs to the point of connection R_5_ with crossvein r-m. Drawings were made based on specimens and the photographs by Iwona Kania-Kłosok. The map was built using the map Maps-For-Free (https://maps-forfree.com) and modified with the software packages Corel Draw and Corel PhotoPaint X7. The wing venation nomenclature is follows Alexander^5^, Krzemiński and Krzemińska^56^, the term “d-cell base” is used after Krzemiński and Freiwald^3^, the designation of the hypopygium were introduced after McAlpine et al.^57^ and Podenas^58^.

### Nomenclatural acts

The electronic edition of this article conforms to the requirements of the amended International Code of Zoological Nomenclature, and hence the new names contained herein are available under that Code from the electronic edition of this article. This published work and the nomenclatural acts it contains have been registered in ZooBank, the online registration system for the ICZN. The ZooBank LSIDs (Life Science Identifiers) can be resolved and the associated information viewed through any standard web browser by appending the LSID to the prefix ‘http://zoobank.org/’. The LSID for this publication is: urn:lsid:zoobank.org:pub: 89897A75-CCFF-45D3-B0DB-0D5B0B73C6C0, for a new species is: urn:lsid:zoobank.org:act: CC518ED7-39ED-4BD9-A0EE-00D05E7F8FD4.

## Supplementary Information

Supplementary Information.

## Data Availability

All data generated or analyzed during this study are included in this published article.

## Abbreviations

GMUG: University of Gottingen
ISEA PAS: Institute of Systematic and Evolution of Animals, Polish Academy of Sciences
NHMB: Natural History Museum Humboldt University, Berlin
NHM: Natural History Museum, London, UK
PIN RAS: Paleontological Institute, Russian Academy of Sciences, Moscow
SDEI: Senckenberg Deutsches Entomologisches Institut
ZMB: Zoological Museum Berlin
