# Supplementary material for: New finding of Toxorhina (Ceratocheilus) limoniid fly in Eocene Baltic amber and the biogeographical context of the genus
Source: Sci Rep. 2022 Nov 12;12:19382. doi: 10.1038/s41598-022-23866-7 (PMC9653493; doi:10.1038/s41598-022-23866-7)
Supplement: Supplementary file 1 — Supplementary Information. [file 41598_2022_23866_MOESM1_ESM.docx]

Supplementary file to:

**New finding of *Toxorhina* (*Ceratocheilus*) limoniid fly in Eocene Baltic amber and the biogeographical context of the genus**

Iwona Kania-Kłosok^1*^, Wiesław Krzemiński^2^, Jacek Szwedo^3^

^1^Department of Biology, Institute of Biology and Biotechnology, University of Rzeszów, Poland; e–mail: ikania@ur.edu.pl ORCID: 0000-0002-2325-4308

^2^Institute of Systematics and Evolution of Animals, Polish Academy of Sciences, Kraków, Poland ORCID: 0000-0001-5685-891X

^3^ Laboratory of Evolutionary Entomology and Museum of Amber Inclusions, Department of Invertebrate Zoology and Parasitology, Faculty of Biology, University of Gdańsk, 59, Wita Stwosza St., PL80-308 Gdańsk, Poland ORCID: 0000-0002-2796-9538

**1. Systematic treatement**

**1.1. Alphabetic list of taxonomic names**

*Ceratocheilus* Wesché, 1910^79^

Diptera Linnaeus, 1758^71^

*Elephantomyia* Osten Sacken, 1860^75^

*Eutoxorhina* Alexander, 1934^60^

*Helius* Lepeletier and Serville, 1828^70^

Limoniidae Speiser, 1909^78^

Limoniinae Speiser, 1909^78^

Tipulomorpha Rohdendorf, 1961^76^

*Toxorhina* (*Ceratocheilus*) *caucasiensis* Krzemiński and Freiwald, 1991^56^

*Toxorhina* (*Ceratocheilus*) *contractifrons* (Edwards, 1933)^64^

*Toxorhina* (*Ceratocheilus*) *eridanus* Meunier, 1917^74^

*Toxorhina* (*Ceratocheilus*) *fulvicolor* Alexander, 1967^61^

*Toxorhina* (*Ceratocheilus*) *mexicana* Kopeć, Kania and Krzemiński, 2016^69^

*Toxorhina* (*Ceratocheilus*) *omnifusca* Zhang, Li and Yang, 2015^6^

*Toxorhina* Loew, 1850^72^

*Toxorhina madagascariensis* Meunier, 1906^73^

**1.2. Systematic treatment and chresonymy**

Order: Diptera Linnaeus, 1758^71^

Infraorder: Tipulomorpha Rohdendorf, 1961^76^

Family: Limoniidae Speiser, 1909^78^

Subfamily: Limoniinae Speiser, 1909^78^

Genus: T***oxorhina*** Loew, 1850^72^

Type species: T*oxorhina fragilis* Loew, 1850^72^: 36, by subsequent designation by Osten Sacken 1860^75^: 113.

*Toxorrhina* auct., error forT *oxorhina*

Subgenus: *Ceratocheilus* Wesché, 1910^79^

Type species: *Ceratocheilus winnsampsoni* Wesché, 1910^79^ [= *Styringomyia cornigera* Speiser, 1908^77^: 130], by subsequent designation of Brunetti 1920^63^: 230.

1910 *Ceratocheilus* Wesché^79^, p. 358 (as genus)

1912 *Neostyringomyia* Alexander^59^, p. 85 (1912) (as subgenus of *Styringomyia*).

1918 *Conithorax* Brunetti^62^, p. 298 (1918) (as genus).

***Toxorhina* (*Ceratocheilus*) *eridanus*** Meunier, 1917^73^

1917 *Ceratocheilus* *eridanus*: Meunier^73^, pp. 96–97, pl. 15, figs 67, 68, pl. 16, fig. 72.

1931 *Ceratocheilus eridanus*: Alexander^54^, p. 124, figs 168, 169.

1982 *Ceratocheilus eridanus*: Keilbach^68^, p. 326.

1994 *Toxorhina* (*Ceratocheilus*) *eridanus*: Evenhuis^65^, p. 87.

2015 *Toxorhina* (*Ceratocheilus*) *eridanus*: Kania^67^, pp. 85, 86, figs 26A-C, 34A-E.

**Supplementary table 1.** Distribution of *Toxorhina* species in zoogeographic regions according to data of Oosterbroek (2022)^2^ and modified zoogeographic divisions of Holt *et al*.^1^ Taxonomic references to species see Oosterbroek (2022). Abbreviations: Nea – Nearctic, Pan – Panamanian, Ntr – Netropic, WAf – West African, EAf – East African, Mad – Madagascan, Ort – Oriental, Oce – Oceanian, Aus – Australian, NZe – Novozealandic.

|  |  | **Nea** | **Pan** | **Ntr** | **WAf** | **EAf** | **Mad** | **Ort** | **Oce** | **Aus** | **NZe** |
| --- | --- | --- | --- | --- | --- | --- | --- | --- | --- | --- | --- |
|  | *Toxorhina* (*Ceratocheilus*) *alexanderi* Tjeder, 1981 |  |  |  | + |  |  |  |  |  |  |
|  | *Toxorhina* (*Ceratocheilus*) *americana* (Alexander, 1913) |  | + | + |  |  |  |  |  |  |  |
|  | *Toxorhina* (*Ceratocheilus*) *approximata* Alexander, 1951 |  |  |  |  |  | + |  |  |  |  |
|  | *Toxorhina* (*Ceratocheilus*) *atritarsis* Alexander, 1943 |  | + |  |  |  |  |  |  |  |  |
|  | *Toxorhina* (*Ceratocheilus*) *australasiae* (Alexander, 1922) |  |  |  |  |  |  |  |  | + |  |
|  | *Toxorhina* (*Ceratocheilus*) *biroi* Alexander, 1934 |  |  |  |  |  |  |  | + |  |  |
|  | *Toxorhina* (*Ceratocheilus*) *bispinosa* Alexander, 1965 |  |  |  |  |  | + |  |  |  |  |
|  | *Toxorhina* (*Ceratocheilus*) *bistyla* Alexander, 1967 |  |  |  |  |  |  | + |  |  |  |
|  | *Toxorhina* (*Ceratocheilus*) *brachymera* Alexander, 1956 |  |  |  |  | + |  |  |  |  |  |
|  | *Toxorhina* (*Ceratocheilus*) *brevifrons* (Brunetti, 1918) |  |  |  |  |  |  | + |  |  |  |
|  | *Toxorhina* (*Ceratocheilus*) *brevisector* Alexander, 1974 |  |  |  |  | + |  |  |  |  |  |
|  | *Toxorhina* (*Ceratocheilus*) *caledonica* Alexander, 1948 |  |  |  |  |  |  |  | + |  |  |
|  | *Toxorhina* (*Ceratocheilus*) *capnitis* Alexander, 1956 |  |  |  |  |  |  | + |  |  |  |
|  | *Toxorhina* (*Ceratocheilus*) *chiapasensis* Alexander, 1938 |  | + |  |  |  |  |  |  |  |  |
|  | *Toxorhina* (*Ceratocheilus*) *claripennis* Alexander, 1958 |  |  |  |  | + |  |  |  |  |  |
|  | *Toxorhina* (*Ceratocheilus*) *cocottensis* Alexander, 1956 |  |  |  |  |  | + |  |  |  |  |
|  | *Toxorhina* (*Ceratocheilus*) *contractifrons* (Edwards, 1933) |  |  |  |  |  |  | + |  |  |  |
|  | *Toxorhina* (*Ceratocheilus*) *cornigera* (Speiser, 1908) |  |  |  | + |  |  |  |  |  |  |
|  | *Toxorhina* (*Ceratocheilus*) *danieleae* Alexander, 1979 |  |  |  |  |  | + |  |  |  |  |
|  | *Toxorhina* (*Ceratocheilus*) *drysdalei* Alexander, 1937 |  |  |  |  |  | + |  |  |  |  |
|  | *Toxorhina* (*Ceratocheilus*) *edwardsi* (Alexander, 1920) |  |  |  | + |  |  |  |  |  |  |
|  | *Toxorhina* (*Ceratocheilus*) *eungellae* Alexander, 1944 |  |  |  |  |  |  |  |  | + |  |
|  | *Toxorhina* (*Ceratocheilus*) *flavicostata* Alexander, 1954 |  |  |  |  |  | + |  |  |  |  |
|  | *Toxorhina* (*Ceratocheilus*) *flavirostris* (Alexander, 1920) |  |  |  | + |  |  |  |  |  |  |
|  | *Toxorhina* (*Ceratocheilus*) *formosensis* (Alexander, 1928) |  |  |  |  |  |  | + |  |  |  |
|  | *Toxorhina* (*Ceratocheilus*) *fulvicolor* Alexander, 1967 |  |  |  |  |  |  | + |  |  |  |
|  | *Toxorhina* (*Ceratocheilus*) *fumipennis* Alexander, 1936 |  |  |  |  |  |  |  | + |  |  |
|  | *Toxorhina* (*Ceratocheilus*) *fuscolimbata* Alexander, 1967 |  |  |  |  |  |  | + |  |  |  |
|  | *Toxorhina* (*Ceratocheilus*) *gilesi* (Edwards, 1911) |  |  |  | + |  |  |  |  |  |  |
|  | *Toxorhina* (*Ceratocheilus*) *gressitti* Alexander, 1962 |  |  |  |  |  |  |  | + |  |  |
|  | *Toxorhina* (*Ceratocheilus*) *growea* Theischinger, 1994 |  |  |  |  |  |  |  |  | + |  |
|  | *Toxorhina* (*Ceratocheilus*) *holvia* Boardman, 2020 |  |  |  | + |  |  |  |  |  |  |
|  | *Toxorhina* (*Ceratocheilus*) *hoogstraali* Alexander, 1948 |  |  |  |  |  |  |  | + |  |  |
|  | *Toxorhina* (*Ceratocheilus*) *huanglica* Zhang, Li and Yang, 2015 |  |  |  |  |  |  | + |  |  |  |
|  | *Toxorhina* (*Ceratocheilus*) *imperatrix* Alexander, 1948 |  |  |  |  |  |  |  | + |  |  |
|  | *Toxorhina* (*Ceratocheilus*) *infuscula* Alexander, 1962 |  |  |  |  |  |  |  | + |  |  |
|  | *Toxorhina* (*Ceratocheilus*) *inobsepta* Alexander, 1978 |  |  |  |  |  |  |  | + |  |  |
|  | *Toxorhina* (*Ceratocheilus*) *juvenca* Alexander, 1948 |  |  |  |  |  |  |  | + |  |  |
|  | *Toxorhina* (*Ceratocheilus*) *kokodae* Alexander, 1951 |  |  |  |  |  |  |  | + |  |  |
|  | *Toxorhina* (*Ceratocheilus*) *latifrons* (Brunetti, 1918) |  |  |  |  |  |  | + |  |  |  |
|  | *Toxorhina* (*Ceratocheilus*) *leucomelanopus* (Enderlein, 1912) |  | + |  |  |  |  |  |  |  |  |
|  | *Toxorhina* (*Ceratocheilus*) *leucostena* Alexander, 1937 |  | + |  |  |  |  |  |  |  |  |
|  | *Toxorhina* (*Ceratocheilus*) *levis* (Hutton, 1900) |  |  |  |  |  |  |  |  |  | + |
|  | *Toxorhina* (*Ceratocheilus*) *luteibasis* Alexander, 1962 |  |  |  |  |  |  | + |  |  |  |
|  | *Toxorhina* (*Ceratocheilus*) *lyrata* Alexander, 1972 |  |  |  | + |  |  |  |  |  |  |
|  | *Toxorhina* (*Ceratocheilus*) *macrorhyncha* Alexander, 1955 |  | + |  |  |  |  |  |  |  |  |
|  | *Toxorhina* (*Ceratocheilus*) *maculipennis* Alexander, 1936 |  |  | + |  |  |  |  |  |  |  |
|  | *Toxorhina* (*Ceratocheilus*) *majus* (Edwards, 1926) |  |  |  |  |  |  | + |  |  |  |
|  | *Toxorhina* (*Ceratocheilus*) *melanomera* Alexander, 1962 |  |  |  |  |  |  |  | + |  |  |
|  | *Toxorhina* (*Ceratocheilus*) *mesorhyncha* Alexander, 1936 |  |  |  |  |  |  | + |  |  |  |
|  | *Toxorhina* (*Ceratocheilus*) *monostyla* Alexander, 1962 |  |  |  |  |  |  | + |  |  |  |
|  | *Toxorhina* (*Ceratocheilus*) *nasus* Theischinger, 1994 |  |  |  |  |  |  |  |  | + |  |
|  | *Toxorhina* (*Ceratocheilus*) *nigripleura* (Alexander, 1920) |  |  |  | + | + |  |  |  |  |  |
|  | *Toxorhina* (*Ceratocheilus*) *nigropolita* Alexander, 1956 |  |  |  |  |  | + |  |  |  |  |
|  | *Toxorhina* (*Ceratocheilus*) *nimbipleura* Alexander, 1963 |  |  |  | + |  |  |  |  |  |  |
|  | *Toxorhina* (*Ceratocheilus*) *niveitarsis* (Alexander, 1922) |  | + |  |  |  |  |  |  |  |  |
|  | *Toxorhina* (*Ceratocheilus*) *nympha* Alexander, 1961 |  |  |  |  |  |  |  | + |  |  |
|  | *Toxorhina* (*Ceratocheilus*) *ochracea* (Edwards, 1923) |  |  |  |  |  |  |  |  |  | + |
|  | *Toxorhina* (*Ceratocheilus*) *omnifusca* Zhang, Li and Yang, 2015 |  |  |  |  |  |  | + |  |  |  |
|  | *Toxorhina* (*Ceratocheilus*) *phaeoneura* Alexander, 1960 |  |  |  |  | + |  |  |  |  |  |
|  | *Toxorhina* (*Ceratocheilus*) *pictipennis* Alexander, 1972 |  |  |  | + |  |  |  |  |  |  |
|  | *Toxorhina* (*Ceratocheilus*) *pollex* Alexander, 1956 |  |  |  |  | + |  |  |  |  |  |
|  | *Toxorhina* (*Ceratocheilus*) *prolongata* Alexander, 1938 |  |  | + |  |  |  |  |  |  |  |
|  | *Toxorhina* (*Ceratocheilus*) *revulsa* Alexander, 1955 |  | + |  |  |  |  |  |  |  |  |
|  | *Toxorhina* (*Ceratocheilus*) *romblonensis* Alexander, 1929 |  |  |  |  |  |  | + |  |  |  |
|  | *Toxorhina* (*Ceratocheilus*) *scimitar* Alexander, 1956 |  |  |  |  | + |  |  |  |  |  |
|  | *Toxorhina* (*Ceratocheilus*) *seychellarum seychellarum* (Edwards, 1912) |  |  |  | + |  | + |  |  |  |  |
|  | *Toxorhina* (*Ceratocheilus*) *seychellarum subimmaculata* Alexander, 1955 |  |  |  |  | + |  |  |  |  |  |
|  | *Toxorhina* (*Ceratocheilus*) *simplicistyla* Alexander, 1967 |  |  |  |  |  |  | + |  |  |  |
|  | *Toxorhina* (*Ceratocheilus*) *streptotrichia* Alexander, 1962 |  |  |  |  |  |  |  | + |  |  |
|  | *Toxorhina* (*Ceratocheilus*) *superstes* Alexander, 1942 |  | + |  |  |  |  |  |  |  |  |
|  | *Toxorhina* (*Ceratocheilus*) *taiwanicola* (Alexander, 1923) |  |  |  |  |  |  | + |  |  |  |
|  | *Toxorhina* (*Ceratocheilus*) *tasmaniensis* (Alexander, 1926) |  |  |  |  |  |  |  |  | + |  |
|  | *Toxorhina* (*Ceratocheilus*) *tenebrica* Alexander, 1961 |  |  |  |  |  |  |  | + |  |  |
|  | *Toxorhina* (*Ceratocheilus*) *tinctipennis* (Alexander, 1930) |  |  |  |  |  |  | + |  |  |  |
|  | *Toxorhina* (*Ceratocheilus*) *toxopeana* Alexander, 1961 |  |  |  |  |  |  |  | + |  |  |
|  | *Toxorhina* (*Ceratocheilus*) *trichopyga* Alexander, 1962 |  |  |  |  |  |  |  | + |  |  |
|  | *Toxorhina* (*Ceratocheilus*) *tuberifera* Alexander, 1966 |  |  |  |  |  |  | + |  |  |  |
|  | *Toxorhina* (*Ceratocheilus*) *univirgata* Zhang, Li and Yang, 2015 |  |  |  |  |  |  | + |  |  |  |
|  | *Toxorhina* (*Ceratocheilus*) *vulsa* Alexander, 1952 |  | + |  |  |  |  |  |  |  |  |
|  | *Toxorhina* (*Ceratocheilus*) *westralis* Theischinger, 1994 |  |  |  |  |  |  |  |  | + |  |
|  | *Toxorhina* (*Ceratocheilus*) *yamma* Theischinger, 2000 |  |  |  |  |  |  |  |  | + |  |
|  | *Toxorhina* (*Eutoxorhina*) *ammoula* Theischinger, 1994 |  |  |  |  |  |  |  |  | + |  |
|  | *Toxorhina* (*Eutoxorhina*) *parasimplex* Hynes, 1988 |  |  |  |  |  |  |  | + |  |  |
|  | *Toxorhina* (*Eutoxorhina*) *simplex* Alexander, 1934 |  |  |  |  |  |  |  | + |  |  |
|  | *Toxorhina* (*Toxorhina*) *acanthobasis* Alexander, 1960 |  |  |  |  |  |  |  | + |  |  |
|  | *Toxorhina* (*Toxorhina*) *acutapex* Alexander, 1972 |  |  |  | + |  |  |  |  |  |  |
|  | *Toxorhina* (*Toxorhina*) *angustilinea* Alexander, 1930 |  |  |  |  |  |  | + |  |  |  |
|  | *Toxorhina* (*Toxorhina*) *atripes* Alexander, 1922 |  | + |  |  |  |  |  |  |  |  |
|  | *Toxorhina* (*Toxorhina*) *basalis* Alexander, 1951 |  |  |  |  |  |  |  | + |  |  |
|  | *Toxorhina* (*Toxorhina*) *basiseta* Alexander, 1978 |  |  |  |  |  |  |  | + |  |  |
|  | *Toxorhina* (*Toxorhina*) *biceps* Alexander, 1931 |  |  |  |  |  |  | + |  |  |  |
|  | *Toxorhina* (*Toxorhina*) *brevirama* Alexander, 1953 |  |  |  |  |  |  | + |  |  |  |
|  | *Toxorhina* (*Toxorhina*) *brevistyla* Alexander, 1965 |  |  |  |  |  | + |  |  |  |  |
|  | *Toxorhina* (*Toxorhina*) *brunniventris* Edwards, 1931 |  |  |  |  |  |  | + |  |  |  |
|  | *Toxorhina* (*Toxorhina*) *carunculata* Alexander, 1970 |  | + |  |  |  |  |  |  |  |  |
|  | *Toxorhina* (*Toxorhina*) *centralis* (Alexander, 1913) |  | + |  |  |  |  |  |  |  |  |
|  | *Toxorhina* (*Toxorhina*) *cisatlantica* Speiser, 1908 |  |  |  |  | + |  |  |  |  |  |
|  | *Toxorhina* (*Toxorhina*) *curtipennis* Alexander, 1975 |  |  |  |  | + |  |  |  |  |  |
|  | *Toxorhina* (*Toxorhina*) *curvata* Alexander, 1938 |  | + |  |  |  |  |  |  |  |  |
|  | *Toxorhina* (*Toxorhina*) *cuthbertsoni* Alexander, 1937 |  |  |  | + |  |  |  |  |  |  |
|  | *Toxorhina* (*Toxorhina*) *dendroidea* Alexander, 1931 |  |  |  |  |  |  | + |  |  |  |
|  | *Toxorhina* (*Toxorhina*) *digitifera* Alexander, 1964 |  |  |  |  |  |  |  | + |  |  |
|  | *Toxorhina* (*Toxorhina*) *distalis* Alexander, 1936 |  | + |  |  |  |  |  |  |  |  |
|  | *Toxorhina* (*Toxorhina*) *domingensis* Alexander, 1937 |  | + |  |  |  |  |  |  |  |  |
|  | *Toxorhina* (*Toxorhina*) *duyagi* Alexander, 1930 |  |  |  |  |  |  | + |  |  |  |
|  | *Toxorhina* (*Toxorhina*) *fasciata* Edwards, 1926 |  |  |  |  |  |  | + |  |  |  |
|  | *Toxorhina* (*Toxorhina*) *flavida* (Alexander, 1913) |  | + |  |  |  |  |  |  |  |  |
|  | *Toxorhina* (*Toxorhina*) *fragilis* Loew, 1851 |  | + |  |  |  |  |  |  |  |  |
|  | *Toxorhina* (*Toxorhina*) *grahami* (Wesche, 1910) |  |  |  | + |  |  |  |  |  |  |
|  | *Toxorhina* (*Toxorhina*) *grossa* Alexander, 1956 |  |  |  |  | + |  |  |  |  |  |
|  | *Toxorhina* (*Toxorhina*) *incerta* Brunetti, 1912 |  |  |  |  |  |  | + |  |  |  |
|  | *Toxorhina* (*Toxorhina*) *infumata* Edwards, 1928 |  |  |  |  |  |  |  | + |  |  |
|  | *Toxorhina* (*Toxorhina*) *infumipennis* Alexander, 1942 |  | + |  |  |  |  |  |  |  |  |
|  | *Toxorhina* (*Toxorhina*) *jamaicensis* Alexander, 1964 |  | + |  |  |  |  |  |  |  |  |
|  | *Toxorhina* (*Toxorhina*) *latamera* Alexander, 1968 |  | + |  |  |  |  |  |  |  |  |
|  | *Toxorhina* (*Toxorhina*) *longicollis* Pierre, 1924 |  | + |  |  |  |  |  |  |  |  |
|  | *Toxorhina* (*Toxorhina*) *magna* Osten Sacken, 1865 | + |  |  |  |  |  |  |  |  |  |
|  | *Toxorhina* (*Toxorhina*) *mashona* Alexander, 1959 |  |  |  | + |  |  |  |  |  |  |
|  | *Toxorhina* (*Toxorhina*) *megatricha* Alexander, 1961 |  |  |  |  |  |  |  | + |  |  |
|  | *Toxorhina* (*Toxorhina*) *mendosa* Alexander, 1935 |  |  | + |  |  |  |  |  |  |  |
|  | *Toxorhina* (*Toxorhina*) *meridionalis* (Alexander, 1913) |  | + |  |  |  |  |  |  |  |  |
|  | *Toxorhina* (*Toxorhina*) *montina* Alexander, 1931 |  |  |  |  |  |  | + |  |  |  |
|  | *Toxorhina* (*Toxorhina*) *muliebris* Osten Sacken, 1865 | + |  |  |  |  |  |  |  |  |  |
|  | *Toxorhina* (*Toxorhina*) *nigrivena* Alexander, 1939 |  | + |  |  |  |  |  |  |  |  |
|  | *Toxorhina* (*Toxorhina*) *noeliana* Alexander, 1956 |  |  |  |  |  |  |  | + |  |  |
|  | *Toxorhina* (*Toxorhina*) *occlusa* Edwards, 1928 |  |  |  |  |  |  | + |  |  |  |
|  | *Toxorhina* (*Toxorhina*) *ochreata* Edwards, 1931 |  |  |  |  |  |  | + |  |  |  |
|  | *Toxorhina* (*Toxorhina*) *pergracilis* Alexander, 1944 |  | + |  |  |  |  |  |  |  |  |
|  | *Toxorhina* (*Toxorhina*) *perproducta* Alexander, 1956 |  |  |  |  |  |  |  | + |  |  |
|  | *Toxorhina* (*Toxorhina*) *phoracaena* Alexander, 1966 |  | + |  |  |  |  |  |  |  |  |
|  | *Toxorhina* (*Toxorhina*) *polycantha* Alexander, 1940 |  | + |  |  |  |  |  |  |  |  |
|  | *Toxorhina* (*Toxorhina*) *polytricha* Alexander, 1970 |  | + |  |  |  |  |  |  |  |  |
|  | *Toxorhina* (*Toxorhina*) *producta* Edwards, 1928 |  |  |  |  |  |  | + |  |  |  |
|  | *Toxorhina* (*Toxorhina*) *protrusa* Alexander, 1962 |  |  |  |  |  |  |  | + |  |  |
|  | *Toxorhina* (*Toxorhina*) *pulvinaria* Alexander, 1950 |  |  |  |  |  |  |  | + |  |  |
|  | *Toxorhina* (*Toxorhina*) *scapania* Alexander, 1966 |  | + |  |  |  |  |  |  |  |  |
|  | *Toxorhina* (*Toxorhina*) *scita* Alexander, 1962 |  |  |  |  |  |  | + |  |  |  |
|  | *Toxorhina* (*Toxorhina*) *serpens* Alexander, 1951 |  |  |  |  |  | + |  |  |  |  |
|  | *Toxorhina* (*Toxorhina*) *sparsiseta* Alexander, 1962 |  |  |  |  |  |  | + |  |  |  |
|  | *Toxorhina* (*Toxorhina*) *staplesi* Alexander, 1973 |  |  |  |  |  |  |  | + |  |  |
|  | *Toxorhina* (*Toxorhina*) *stenomera* Alexander, 1972 |  |  |  | + |  |  |  |  |  |  |
|  | *Toxorhina* (*Toxorhina*) *stenophallus* Alexander, 1937 |  | + | + |  |  |  |  |  |  |  |
|  | *Toxorhina* (*Toxorhina*) *subfragilis* Alexander, 1970 |  | + |  |  |  |  |  |  |  |  |
|  | *Toxorhina* (*Toxorhina*) *suttoni* Alexander, 1936 |  |  |  |  |  |  |  | + |  |  |
|  | *Toxorhina* (*Toxorhina*) *taeniomera* Alexander, 1950 |  |  |  |  | + |  |  |  |  |  |
|  | *Toxorhina* (*Toxorhina*) *tonkouiana* Alexander, 1958 |  |  |  |  | + |  |  |  |  |  |
|  | *Toxorhina* (*Toxorhina*) *trichorhyncha* Edwards, 1926 |  |  |  |  |  |  | + |  |  |  |
|  | *Toxorhina* (*Toxorhina*) *trilineata* Alexander, 1936 |  |  |  |  |  |  |  | + |  |  |
|  | *Toxorhina* (*Toxorhina*) *trilobata* Alexander, 1938 |  | + |  |  |  |  |  |  |  |  |
|  | *Toxorhina* (*Toxorhina*) t*uberculata* Alexander, 1931 |  |  |  |  |  |  | + |  |  |  |
|  | *Toxorhina* (*Toxorhina*) *violaceipennis* Alexander, 1937 |  | + |  |  |  |  |  |  |  |  |
|  | *Toxorhina* (*Toxorhina*) *westwoodi* Brunetti, 1920 |  | + | + |  |  |  |  |  |  |  |

**Complementary references**

59. Alexander, C.P. New African Tipulidae. *Canadian Entomologist* **44**, 83–88 (1912).

60. Alexander, C.P. New or little-known Tipulidae from eastern Asia (Diptera). XVIII. Philippine Journal of Science 53, 267–300 (1934).

61. Alexander, C.P. Undescribed species of crane flies from the Himalaya mountains (Diptera: Tipulidae). XV. *Journal of the New York Entomological Society* **75**, 183–187 (1967).

62. Brunetti, E. Revision of the Oriental Tipulidae with descriptions of new species, Part 2. *Records of the Indian Museum* **15**, 255–344 (1918).

63. Brunetti, E. Catalog of Oriental and south Asiatic Nemocera. *Records of the Indian Museum* **17**, 1–300 (1920).

64. Edwards, F.W. Diptera Nematocera from Mount Kinabalu. *Journal of the Federated Malay States Museums* **17**, 223–296 (1933).

65. Evenhuis, NL. Catalogue of the fossil flies of the world (Insecta: Diptera). Backhuys, Leiden, 1–600 (1994).

66. Hutson, A.M. *Toxorhina madagascariensis* Meunier. Systema Dipterorum, Version 3.9. 1 record. http://sd.zoobank.org/Nomenclator/Details/184292, accessed on 20.07.2022 (1980).

67. Kania, I. The taxonomy of the selected genera of the subfamily Limoniinae (Diptera: Limoniidae) from Baltic amber (Eocene), with notes on their phylogeny. *Annales Zoologici* **65**(1), 71–100 (2015).

68. Keilbach R. Bibliographie und Liste der ArtenTierischer Einschlusse in fossilen Harzen sowie ihrer Aufbewahrungsorte. *Deutsche Entomologische Zeitschrift* **29(4-5)**, 301–-491 (1982).

69. Kopeć, K., Kania, I. & Krzemiński, W. New and little known crane-fly species ofThe genera *Helius*, *Elephantomyia* and *Toxorhina* (Diptera, Limoniidae) from Dominican and Mexican amber. *Palaeontologia Electronica* **19.2.26A**, 1–14 (2016).

70. Lepeletier, A.L.M. & Serville, J.G.A. Entomologie, ou histoire naturelle des crustacés, des arachnides et des insectes. *Encyclopédie méthodique. Histoire naturelle des animaux* **10**, 345–833 (1828).

71. Linnaeus, C. *Systema nature per regnaTria naturae, secundum classes, ordines, genera, species, cum caracteribus, differentiis, synonymi, locis. Tomus I.* Editio decima, reformata. L. Salvii, Holmiae [= Stockholm] pp. 824 (1758).

72. Loew, H. Uber den Bernstein Und die Bernsteinfauna. *Program der Keiserischen Realschule Meseritz*, 44 pp. (1850).

73. Meunier, F. Sur une nouvelle espèce deT*oxorrhina* du copal récent de Madagascar. *Bulletin Société d'étude des sciences naturelles et du Musée d’Elbeuf* **24**, 97–100 (1906).

74. Meunier, F. Ueber einige Mycetophiliden und Tipuliden des Bernsteins nebst Beschreibung der Gattung *Palaeotanypeza* (Tanypezinae) derselben Formation. *Neues Jahrbuch für Mineralogie, Geologie und Paläontologie* **1917(3)**, 73–106 (1917).

75. Osten Sacken, C.R. New genera and species of North American Tipulidae with short palpi, with an attempt at a new classification of the tribe. *Proceedings of the Academy of Natural Sciences of Philadelphia* 1859, 197–254 (1860).

76. Rohdendorf, B.B. The oldest infraorders of Diptera from the Triassic of Middle Asia. *Palaeontologicheskoi Zhurnal* **2**, 90–100 (1961).

77. Speiser, P. Dipteren aus Deutschlands afrikanischen Kolonieen. *Berliner entomologische Zeitschrift* **52**, 127–149 (1908).

78. Speiser, P. 4 Orthorapha. Orthorapha Nematocera. *Wissenschaftliche ergebnisse der Schwedischen zoologischen expedition nach dem Kilimandjaro, dem Meru und den umgebenden Massaisteppen Deutsch-Ostafrikas 1905–1906, unter leitung von Prof. Dr. Yngve Sjostedt. (Diptera)* **10**, 31–65 (1909).

79. Wesché, W. On the new Tipulid subfamily Ceratocheilinae. *Journal of the Linnean Society of London,* *Zoology* **30**, 355–360 (1910).
